# Impact of the surgical strategy on the incidence of C5 nerve root palsy in decompressive cervical surgery

**DOI:** 10.1371/journal.pone.0188338

**Published:** 2017-11-16

**Authors:** Theresa Krätzig, Malte Mohme, Klaus C. Mende, Sven O. Eicker, Frank W. Floeth

**Affiliations:** 1 Department of Neurosurgery, University Medical Center Hamburg-Eppendorf, Hamburg, Germany; 2 Department of Spine Surgery, Hospital zum Heiligen Geist, Kempen, Germany; Seoul National University College of Medicine, REPUBLIC OF KOREA

## Abstract

**Objective:**

Our aim was to identify the impact of different surgical strategies on the incidence of C5 palsy.

**Background:**

Degenerative cervical spinal stenosis is a steadily increasing morbidity in the ageing population. Postoperative C5 nerve root palsy is a common complication with severe impact on the patients´ quality of life.

**Methods:**

We identified 1708 consecutive patients who underwent cervical decompression surgery due to degenerative changes. The incidence of C5 palsy and surgical parameters including type and level of surgery were recorded to identify predictors for C5 nerve palsy.

**Results:**

The overall C5 palsy rate was 4.8%, with 18.3% of cases being bilateral. For ACDF alone the palsy rate was low (1.13%), compared to 14.0% of C5 palsy rate after corpectomy. The risk increased with extension of the procedures. Hybrid constructs with corpectomy plus ACDF at C3-6 showed significantly lower rates of C5 palsy (10.7%) than corpectomy of two vertebrae (*p* = 0.005). Multiple regression analysis identified corpectomy of C4 or C5 as a significant predictor. We observed a lower overall incidence for ventral (4.3%) compared to dorsal (10.9%) approaches (*p*<0.001). When imaging detected a postoperative shift of the spinal cord at index segment C4/5, palsy rate increased significantly (33.3% vs. 12.5%, *p* = 0.034).

**Conclusions:**

Extended surgical strategies, such as dorsal laminectomies, multilevel corpectomies and procedures with extensive spinal cord shift were shown to display a high risk of C5 palsy. The use of extended procedures should therefore be employed cautiously. Switching to combined surgical methods like ACDF plus corpectomy can reduce the rate of C5 palsy.

## Introduction

C5 nerve root palsy is a common complication in cervical spine surgery with an overall prevalence of 5–6%, with ranges from 0–26.4% for anterior and 0–50% for posterior procedures[1,2]. It is defined as a mostly unilateral severe weakness of the deltoid and a concomitant mild to moderate weakness of the biceps muscle with additional sensory loss and pain in the C5 dermatome, present in 50% of cases[3,4]. The symptoms are exclusively (mono)-radicular without myelopathic features and the onset may vary from hours to weeks postoperatively[2,3,5]. Although postoperative C5 nerve root palsy generally has a good prognosis with a spontaneous full recovery in most of the cases within the first six months after surgery, it has a severe impact on the patients’ rehabilitation and quality of life. Patients with more severe and bilateral palsy or loss of somatic sensation with pain, however, require significantly longer recovery times of up to two years[2,3,5–7].

Over the last decade, many efforts have been made to understand, foresee and avoid this complication. Unfortunately, the origin of postoperative C5 nerve root palsy is still not clearly understood. Most authors suspect stretching of the C5 nerve root and tethering inside the neuroforamen, partially due to posterior shift of the spinal cord after a dorsal decompression procedure, as the main cause[2,8]. A pre-existing subclinical C5 nerve root compression caused by a narrow C4/5 foramen might also be seen as a reason for postoperative C5 nerve root palsy[4,9–12]. Other theories include excessive intraoperative traction[13,14], spinal cord ischemia due to decreased blood supply from radicular arteries[9], reperfusion injury of the spinal cord[15,16], an inadvertent injury to the nerve root during surgery[17] or preoperative spinal cord rotation as factors causing postoperative C5 nerve root palsy[18].

The aim of our study was to analyze and identify predictors of C5 nerve root palsy in, to the best of our knowledge, one of the largest cohorts to date in order to detect potential pitfalls and gain new insights into recommendations for the surgical strategy of cervical decompressions. We hypothesized that a differentiated analysis of the extent and level of surgery can aid in determining the pathophysiological basis for C5 nerve root palsy.

## Materials and methods

### Patient cohort

We prospectively identified the C5 palsy rate in all patients receiving cervical decompression surgery due to degenerative central cervical stenosis with myelopathy and/or lateral neuroforaminal stenosis with radiculopathy between 11/2008 and 12/2015. Clinical parameters of patients with cervical stenosis were prospectively identified. Data analysis was performed retrospectively on anonymized datasets. The study was conducted in accordance with ethical guidelines of the local ethical review board of the Hamburg chamber of physicians, Hamburg, Germany (IRB number WF-09/17). All procedures performed in studies involving human participants were in accordance with the ethical standards of the 1964 Helsinki Declaration. All surgeries were performed by a single surgeon (FWF) at a single institution. Patients with ossification of the posterior longitudinal ligament (OPLL), tumor, traumatic lesions or infectious diseases of the spine were excluded.

### Surgical indication strategy and technique

Anterior approaches (n = 1589) were performed when the compression was predominantly located ventrally of the spinal cord and the number of involved disc segments was restricted to two or maximum of four levels (i.e. mono-, bi- or trisegmental stenosis).

ACDF up to three levels was regarded as the standard anterior decompressive procedure when a decompression of the spinal canal and/or the neuroforamen was sufficiently achievable through the involved disc spaces only. Bilateral foraminotomy for neurolysis of the exiting nerve roots was performed using a drill and a 1–2 mm punch.

**Fig 1:**
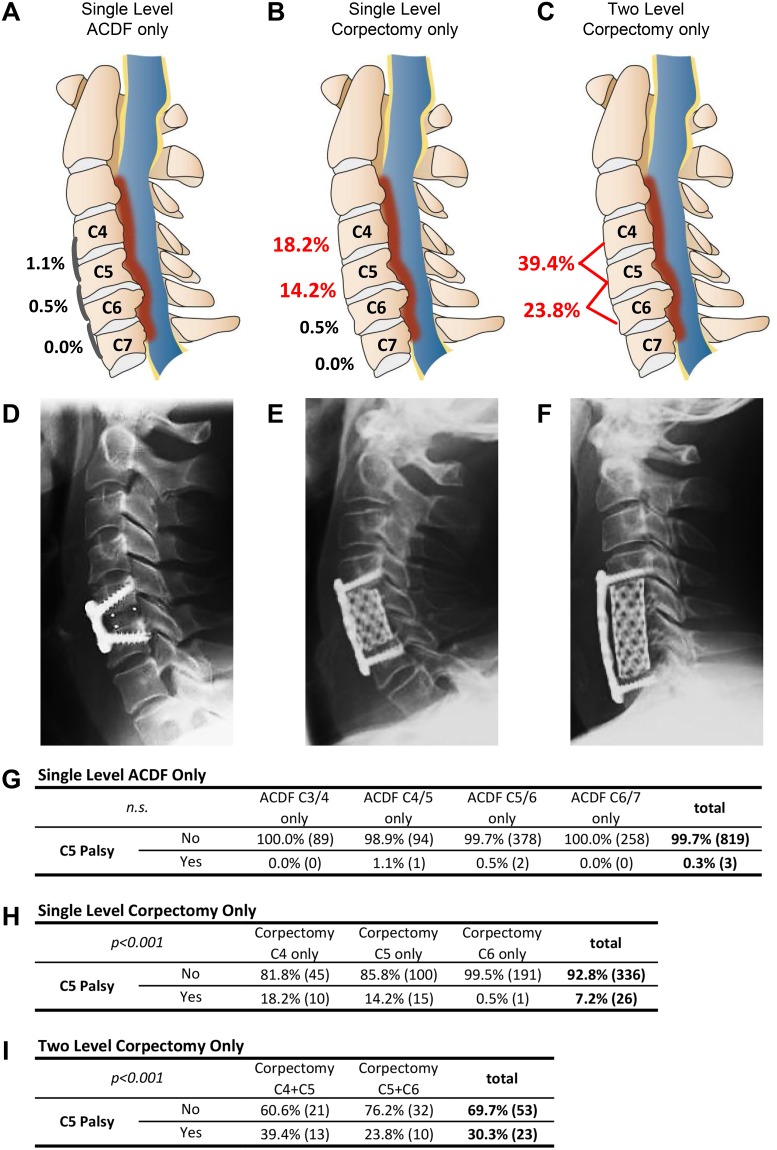
Schematic overview of C5 palsy rate for single level ACDF (A), single level corpectomy (B) or two level corpectomy (C) for the respective cervical level (not all levels shown). The cervical spine is shown in the sagittal view with the spinal cord marked in blue, the surrounding cerebrospinal fluid in yellow and a schematic stenotic narrowing in red. D-F shows representative corresponding lateral X-ray images of the surgical results. Single level ACDF (D), single level corpectomy (E) and two level corpectomy (F). Integrated tables G-I display the additional statistical values to the corresponding surgical strategies, including the overall C5 palsy rate for the respective surgical approach. Statistical analysis applied routine cross tables and chi^2^ testing.

A corpectomy with complete removal of the vertebral body with its adjacent discs including the posterior ligament was performed when the goal of decompression of the spinal canal and/or the neuroforamen did not seem achievable through the involved disc spaces only. Replacement of the vertebral body was performed using a titanium mesh cage (10 to 16 mm diameter) with an additional fixation with a ventral titanium plate (Fig 1E).

We performed a posterior decompression (n = 119) in cases in which the stenosis was predominantly located dorsally and when multilevel decompression of more than three segments was necessary, except in cases with additional kyphotic degenerative deformity, in which a ventral approach was chosen. A complete laminectomy was performed in all involved segments with undercutting of the adjacent segments followed by stabilization using a screw-rod system. Lateral mass screws were used in C3-6 and pedicle screws in C2, C7, T1, and T2.

### Statistical analysis

IBM SPSS v22^®^ and the R Foundation’s R v2.12 were used for statistical analysis. Routine cross tables, chi^2^ and t-tests were used for baseline data evaluation. Significant impactors for C5 nerve root palsy were identified using backwards multivariate nominal regression analysis, validated by forward regression. Graphical analysis was performed using Systat Sigmaplot^®^ v13.0 and Microsoft^™^ Excel 2010^®^.

## Results

### Patient characteristics

We included a total of 1708 patients, 1057 with ACDF only (1322 segments), and 466 with corpectomy (639 vertebrae). In 66 cases a combination of corpectomy and ACDF was applied and 119 patients were treated with dorsal decompression and instrumentation (529 pairs of screws). Overall, 54.5% of patients were female and 45.5% were male, with a mean age of 61 years (SD ±11.6, range 24–89 years).

### Surgical strategy and C5 nerve root palsy

The overall rate of C5 palsy, irrespective of the approach chosen, was 4.8% (n = 82/1708 cases). Bilateral involvement was observed in 18.3% (n = 15/82) of those C5 palsies (0.9% overall incidence of bilateral palsy). For ACDF, the overall rate of postoperative C5 palsy was 1.13% (n = 12/1057), and in combination with corpectomy palsy rate was 9.1% (n = 6/66). The rate of C5 nerve palsy for all levels after corpectomy without the combination of ACDF was 10.9% (n = 51/466). If only dorsal decompression and instrumentation was employed, the rate of C5 palsy was 10.9% (n = 13/119). If an isolated ventral approach was taken the palsy rate was 4.3% (n = 68/1580), for an isolated dorsal approach, it was 10.9% (n = 13/119) and in dorso-ventral procedures, C5 palsy accounted for 11.1% (n = 1/9, *p*<0.001).

For single segment ACDF, 0.35% (n = 3/881) developed a C5 palsy (C4/5 1.1% and C5/6 0.5%, Fig 1A and 1G), which was 3.75% (n = 6/160) of patients with a two segment approach (C3-5 25.0% and C4-6 11.1%), and 18.8% of (n = 3/16) of patients with a three segment approach (C4-7). The occurrence of C5 nerve root palsy was significantly higher if more levels were involved in the fusion process (*p*<0.001). No paresis was be observed if the segments C4/5 and/or C5/6 were not included in the procedure.

Patients with corpectomy and without dorsal instrumentation presented with an overall rate of C5 nerve root palsy of 11% (n = 51/466, *p*<0.001). For corpectomy of a single vertebra the overall rate was 6.5% (n = 24/370); if two vertebrae were resected, the rate significantly increased to 28.1% (n = 25/89, *p*<0.001). In three level corpectomy, 20% C5 palsy was found (n = 1/5), reaching 50% in four level corpectomy (n = 1/2). If corpectomy of the C4 vertebra only was performed, the rate of C5 palsy was 18.2% (n = 10/55), compared to 14.2% (n = 15/115) for C5, and 0.5% (n = 1/192, *p*<0.001) for C6 corpectomy only (Fig 1B and 1H). However, if the surgical approach involved two vertebrae, the C5 palsy rate markedly increased to 39.4% for C4+C5 corpectomy and to 23.8% for C5+6 (*p*<0.001) (Fig 1C and 1I).

**Fig 2:**
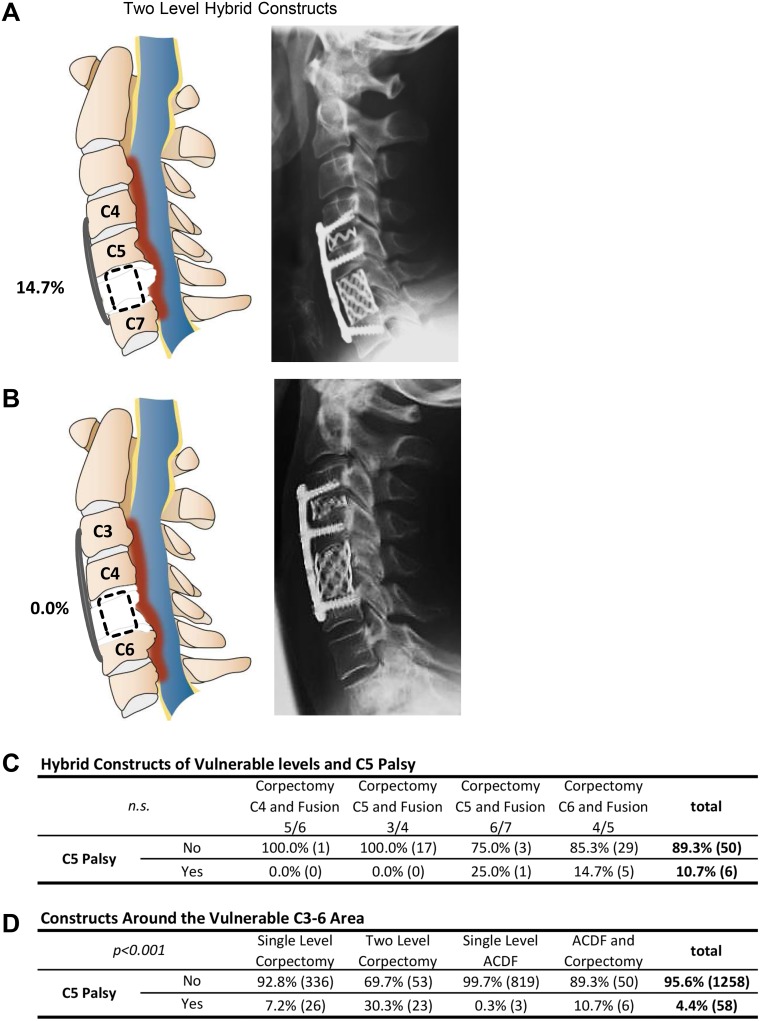
Sagittal schematic of the surgical spine to illustrate C5 palsy rate of two level hybrid constructs, i.e. combination of corpectomy and ACDF, for the levels C4-7 (A) and C3-6 (B). Statistical values are summarized in C, including overall C5 incidence rate for hybrid constructs on all levels around the vulnerable level of C4/C5. Additional information on other possible surgical constructs around the level of C4/C5 is presented in D, allowing for the direct comparison of C5 palsy rate of the different surgical strategies. Statistical analysis applied routine cross tables and chi^2^ testing.

In three segment approaches concerning the “vulnerable” area around the C4/5 segment, different options were compared. C5 corpectomy combined with ACDF for C3/4 and C4 corpectomy with ACDF for C5/6 did not present any C5 palsies (n = 0/17 and n = 0/1). C5 corpectomy combined with ACDF on C6/7 showed 25% palsy (n = 1/3), while corpectomy of C6 in combination with ACDF for C4/5 presented a C5 palsy rate of 14.7% (n = 5/29, *p* = n.s.) (Fig 2C).

If dorsal instrumentation and decompression without additional ACDF or corpectomy was performed the overall C5 palsy rate was 10.9%. C5 palsy was present in 5% (n = 1/20) for one segment, 5.6% (n = 1/18) for two segments, 6.3% (n = 1/16) for three segments, 17.4% (n = 4/23) for six segments, and 20.0% (n = 2/10) for seven segments with laminectomy and dorsal instrumentation. The segment C4/5 was included in all but 12 of those procedures. These 12 cases presented no C5 palsy. The remaining 107 patients with inclusion of the C4/5 segment demonstrated a C5 palsy rate of 15.1% (n = 13/107).

**Fig 3:**
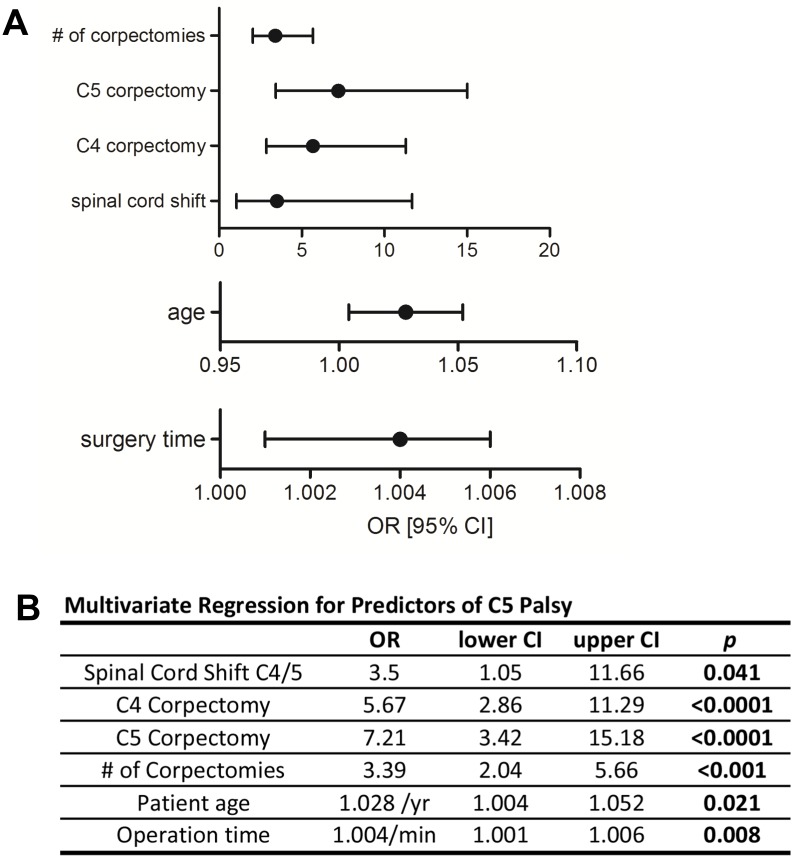
Multivariate regression analysis of predictive outcome parameters for C5 palsy rate as illustrated by forest plots (A). Dots indicate the odds-ratio (OR) and whiskers for 95% confidence interval (CI). Integrated table (B) shows the corresponding statistical values including the p-value after regression analysis.

Multiple regression analysis of corpectomy involving C2-T2 identified surgery at the levels C4 (OR 5.67, 95% CI: 2.86–11.29, p<0.0001) and C5 (OR 7.21, 95% CI: 3.42–15.18, *p*<0.0001) as significant predictors regarding C5 palsy (Fig 3). The number of corpectomies was also highly associated with the incidence of C5 palsy (OR 3.39, 95% CI: 2.04–5.66, *p*<0.001) (Fig 3). In conjunction with the above-presented data on ACDF, with only 2.6% of cases when corpectomy of C6 was performed, C5 paresis almost exclusively occurred if the corpectomy involved vertebrae C4 and C5.

### Additional predictive risk factors

Overall, stenosis of multiple segments was correlated with an increased rate of C5 palsy. C5 nerve root paresis was observed if one, two, three, four or five segments were stenotic in 1% (n = 9/895), 6% (n = 33/529), 14% (n = 30/210), 16% (n = 8/49), and 18% (n = 2/11), respectively (*p<*0.001). No C5 palsy was seen in patients with stenosis of more than 5 segments. In cases with isolated ventral stenosis, no C5 palsy occurred, while in cases with isolated dorsal stenosis, 13% presented with a postoperative C5 nerve palsy. Furthermore, circumferential stenosis resulted in a C5 palsy rate of 18.2% (*p* = 0.31, n.s.).

**Fig 4:**
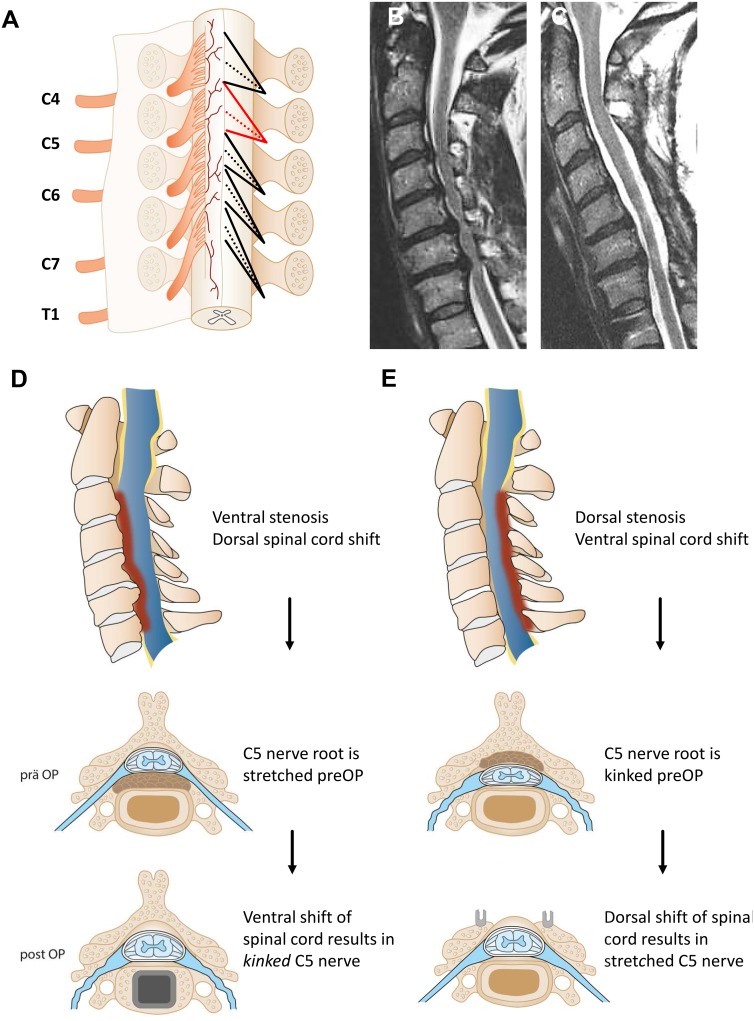
Illustration of the cervical spinal nerve roots exiting the spinal cord (left). The right side schematically depicts the angle, including the superior and inferior rootlets of the individual nerve root, emphasizing the distinct nerve root exit of C5 as formerly described by Hung *et al*. and Alleyne *et al*. [19,20]. Sagittal T2w magnetic resonance imaging of the cervical spine before (B) and after (C) dorsal decompression. This image highlights the dorsal shift of the spinal cord due to decompression. Figure D and E schematically explain the postulated hypothesis on the pathophysiological basis of C5 nerve root palsy. A slowly established ventral stenosis (D) results in a dorsal shift of the myelon, leading to a stretched C5 nerve root (blue), as depicted in the axial view. Ventral decompression through ACDF consecutively leads to a ventral shift of the spinal cord with kinking of the nerve root. A dorsal stenosis (E) represents an analogous situation with dorsal shifting of the spinal cord after laminectomy and fusion, as suggested by the screws. Additional information on the pathophysiological basis can be found in supporting information S1 Video.

Subgroup analysis revealed, that if a shift of the spinal cord at the index segment C4/5 was detected on postoperative radiographic imaging, the C5 palsy rate increased from 12.5% (n = 9/72) if no shift was present to 33.3% (n = 6/18, OR = 0.29, 95% CI 0.09–0.95, *p* = 0.034). A dorsal shift was associated with C5 palsy in 41.7% of cases, while on the contrary only 16.7% of patients with a ventral shift of the spinal cord presented with C5 paresis (*p* = 0.29, n.s.). Interestingly, if a shift greater than 5mm was detected, postoperative C5 paresis was observed in all patients, but only in 7.7% of patients with a shift less than 5mm (*p* = 0.007) (see schematic explanation of pathophysiology, Fig 4 and S1 Video).

For the overall patient collective, age (OR 1.028/year, *95*% CI: 1.004–1.052/year, *p* = 0.021) and operation time (OR 1.004/min, 95% CI: 1.001–1.006/min, *p* = 0.008) were identified as independent predictors for the occurrence of C5 palsy.

### Clinical outcome

Overall, 57.1% of the 82 patients with C5 palsy recovered fully within the time of follow-up period (mean 7.6 months SD 7.3 months range <1–36 months). Due to incomplete data acquisition, not all patient information is available (n = 71/82). However, investigating this subgroup, if a C5 paresis occurred at day one after surgery (n = 61), restitution was very likely (67.9%) compared to a delayed onset of palsy (>1 day, n = 10), with a recovery rate of 14.3% (OR 12.67, 95% CI 1.32–121.47, *p* = 0.010). Patients with a paresis graded 3/5 or 4/5 in manual muscle testing (MMT) experienced full restitution in 75% of all cases compared to 42.1% if the muscle strength was graded 2 or less (*p* = 0.15).

## Discussion

Our study is one of the largest series to date analyzing postoperative C5 nerve root palsy in a prospective survey. Dissecting the influence of different surgical strategies on the prevalence of C5 palsies, we demonstrate that C5 nerve root palsy almost exclusively occurred if vertebrae C4 and/or C5 are included in the surgical strategy. Most significantly, the incidence of C5 paresis increased with the number of segments included in the decompression procedure and the resulting dorsal or ventral shift of the spinal cord. In anterior multilevel approaches hybrid constructs with ACDF and corpectomy were less likely to present with C5 paresis than multilevel corpectomies.

We evaluated patients in which decompression surgery in the cervical spine was indicated due to symptomatic degeneration with stenosis and compression of the spinal cord or neuroforaminal stenosis with compression of the nerve roots was indicated. All other pathologies were excluded in order to reduce influencing variables on the surgical strategies. We consequently analyzed patients with an isolated postoperative C5 paresis, which we believe provides a better baseline for interpreting our results in the context of pathogenesis.

Our overall C5 palsy rate independent of surgical procedures and approaches was 4.8%, which is in line with the incidence of C5 palsy in other large cohorts and reviews after cervical decompression surgery[3,10,21]. Interestingly, bilateral C5 paresis was present less frequently (0.9%) compared to previous studies (5–7%)[11,22], although the lack of laminoplasties in our study, where an asymmetric decompression occurs more often than in laminectomy procedures, would rather suggest a higher rate of bilateral palsy. Isolated ventral stenosis showed the lowest incidence of C5 nerve root palsy, followed by an isolated dorsal stenosis and circumferential stenosis with 0%, 13% and 18.2%, respectively, independent of the following surgical procedure.

In our patient cohort, C5 nerve root palsy rate for ACDF only was lowest with 1.13% which is consistent with the literature[23]. C5 palsy only occurred when segments C4/5 and/or C5/6 were involved in the procedure. With multiple ACDF levels operated on, there was a significant increase of C5 paresis; however, the incidence was still lower than in other multilevel decompression procedures.

In general, C5 palsy rate was found to be much higher with increasing complexity of surgery, as corpectomies displayed a more than 10-times higher rate of C5 paresis. It is important to note that this risk was almost exclusively restricted to C4 or C5 corpectomies. In the two-level procedure, the incidence rose to 39.4% (C4+5). This observation is in line with the study groups of Bydon and Nassr *et al*., who also describe an increasing incidence of C5 palsy after multilevel decompression procedures[21,22]. As the risk of developing a C5 nerve root palsy increased significantly with an increasing number of corpectomies, previous groups suggested the preferential application of a hybrid construct with corpectomy and ACDF whenever possible[23–25]. We now confirm these suggestions, as the overall incidence of C5 palsy in a combination of ACDF with one-level corpectomy was only 10.7% in our patients compared to 30.3% in two-level corpectomy. This can be explained due to a less radical anterior decompression, prohibiting extensive forward shifting of the spinal cord. However, in our series C5 nerve root palsy could only be detected when ACDF C4/5 with corpectomy of C6 was performed (14.7%). In cases with corpectomy of C5, we hypothesize that shifting of the spinal cord in the vulnerable region was less pronounced due to the remaining cranial and caudal vertebrae. When C5 nerve root palsy occurs in a corpectomy of C6 combined with ACDF at C4/5, foraminotomy of that segment might not have been sufficient and a compression of the C5 nerve can even be caused due to a minor shift of the spinal cord at the corpectomy level.

The increased C5 palsy rate with more complex procedures was also reflected in the multivariate regression analysis for independent predictors of C5 palsy. Advanced age and prolonged surgery duration were found to be significant determinants for an increased relative risk to develop C5 palsy. This finding indicates that intraoperative stress and/or the potentially limited ability to compensate for nerve root tension in elderly patients, i.e. due to decreased microvascular integrity as a result of arteriosclerosis, might predispose for nerve root injury[26,27].

Our dorsal approaches only included laminectomy with decompression and fusion. They display an overall postoperative palsy rate of 10.9%. Although a rate up to 24% can be observed, most studies reported similar numbers[7,22]. However, most of these studies are not comparable to each other. In the recent review of Basaran and colleagues only seven other groups over the last 45 years analyzed only posterior laminectomy and fusion as posterior decompression procedure; four of those excluded OPLL as an indication for surgery[23]. Only two retrospective studies were comparable to ours, as one showed a doubled palsy rate of 23% after posterior laminectomy and fusion and interpreted excessive intraoperative traction with a significantly greater change in cervical curvature index as the main reason[14]. In our study, we did not need to change the curvature as much due to the decision to operate on all kyphotic cervical spine deformities from an anterior approach, which might be the reason for the lower numbers of C5 palsies. The other large study of Bydon *et al*. had a similar incidence of 8.6% palsies after posterior laminectomy and fusion[21].

As a large review recently stated, there is no overall statistical difference between anterior or posterior approaches, although some groups describe a significantly higher incidence in posterior procedures[21,28], as reported here. Nevertheless, considering all of the results of the larger studies, there is a trend towards the anterior approach surgeries to represent the safer strategy referring to C5 palsy rates compared to posterior procedures[23]. More important however, the strongest predictor for postoperative C5 nerve root palsy in our study was the extension of the procedure, regardless of whether anterior or posterior decompression was performed.

The pathophysiological basis for the occurrence of C5 nerve root palsy is still not completely understood. A study by Yang *et al*. investigated spinal cord shift after dorsal decompression[29]. They described that C5 palsy rates that were significantly higher after dorsal decompression procedures when there was greater change in dural sac area and a greater spinal cord shift[29]. Another large study also found a significantly greater shift of the spinal cord at C4/5 in the palsy group[11]. We confirm this observation and further demonstrate that a shift of more than 5mm was a highly significant predictor for postoperative C5 paresis as it was observed in all of those patients. The direction of the spinal cord shift, however, did not make a significant difference. The group of Lubelski *et al*. created a “prediction formula” describing a significant decrease of C5 palsy rate for every millimeter increase of anteroposterior- and foraminal diameter by 69% and 98%, respectively. Every degree increase of cord-lamina angle results in a significant increase of C5 palsy by 43%[30]. We thereby emphasize that preoperative evaluation of radiological findings and especially the spinal cord shift in anterior-posterior direction is of great importance to assess the risk of postoperative C5 palsy. This finding illustrates why a combination of ACDF with corpectomy is safer than a multilevel corpectomy as the level of ACDF results in a decreased shift compared to the removal of a complete vertebra.

Since the shift of the spinal cord has a profound impact on C5 palsy rate, it is believed, that the unique anatomy of the C5 roots is crucial for pathogenesis. Cadaver studies showed that the C5 ventral rootlets are shorter and exit more obtusely from the spinal cord[20,31]. C5 superior dorsal rootlets angle significantly less inferiorly from the cervical cord than the other dorsal cervical roots[19]. These distinct anatomic characteristics cause a greater vulnerability to the shift of the spinal cord due to decompression surgery, which is in line with our data (see schematic explanation of pathophysiology, Fig 4 and S1 Video). Anatomically, C4 nerve rootlets have almost the same characteristics as the C5 nerve root which results in a comparable vulnerability. However, a paresis of the diaphragm is not detected as often since it is mostly clinically not apparent, whereas C5 represents a nerve root which results in clinically obvious impairments.

Combining the observation of an anatomically distinct nerve root angle and the significant impact of spinal cord shift on C5 palsy rate points to a potentially decisive role of the neuroforamen. In order to minimize the risk of C5 nerve root stress due to spinal cord shift, additional decompression of the neuroforamen can be considered. Several studies demonstrate that preoperative anteroposterior diameter of the neuroforamen was found to be significantly smaller in patients with C5 palsy[7,11,25]. As a consequence, when a wide decompression, which includes more than one level or a corpectomy with potential excessive shifting of the spinal cord is required, prophylactic foraminotomy at the level C4/5 might reduce C5 nerve root palsy, as we performed in all of our procedures in which the segment C4/5 was involved. In contrast, Bydon *et al*. stated in their large retrospective study a higher risk for C5 palsy after dorsal foraminotomy of C4/5 with an incidence of 14.5%[21]. Here, a contrary situation can be discussed with dorsal shifting of the C5 nerve root with constant position of the spinal cord but equal effect. Depending on the narrowing of the neuroforamen, additional intraoperative neuromonitoring can be considered for at risk cases. Studies showed that modification of intraoperative neuromonitoring with additional intraoperative deltoid and biceps TceMEPs and spEMG can significantly decrease the incidence of C5 nerve root palsy[23,32–34]. Intraoperative neuromonitoring and changes in C5 EMG alerted the surgeon for potential stress of the C5 nerve root, resulting in adaptation of the surgical procedure[32].

C5 nerve root palsy generally has a good prognosis, and in most cases there is a full recovery within six months after the operation[35]. In our study, we found that recovery was dependent on the grade of the paresis and the time of onset postoperatively. Overall duration until full recovery with a mean time of 7.6 months was slightly longer in our study than previously described[5,7,33,35]. Although, it is unknown whether revision surgery can improve outcome, we are in line with previous studies proposing that conservative treatment with neurotrophic drugs, hyperbaric oxygen therapy and especially functional exercise is the treatment of choice[5,35].

## Conclusions

Extensive cervical decompression surgery independent of the chosen approach is at high risk of postoperative C5 nerve root palsy with a severe impact of the patients’ quality of life. We demonstrate that whenever possible, combination of ACDF with corpectomy should be preferred over a multilevel corpectomy in anterior decompression procedures. Avoidance of extensive shifting of the spinal cord is crucial as this appeared to be the pathophysiological basis for C5 nerve root palsy. Taken together, we highlight the importance of preoperative evaluation of radiological images for patients at risk of extensive spinal cord shift. In addition, if the C5 nerve roots appear to be stressed intraoperatively due to spinal cord shift, prophylactic foraminotomy should be performed.

## Supporting information

S1 Video Hypothesis of the pathophysiological basis of C5 nerve root palsy after cervical spinal decompression surgery. At first, the cervical spine is shown anteriorly before the schematic illustration highlights the distinct angle in which the C5 nerve root exits the spinal cord (0:09s). The lateral view then demonstrates how the spinal cord is shifted backwards due to the ventral stenosis and vice versa (0:13s–0:20s). The following axial view demonstrates the simultaneous mechanical stress which is exerted on the nerve root during stenosis development (0:23s–0:28s) and after decompressive surgery (0:30s–0:40s). It is important to note that the sudden change in spinal cord shift is suspected to be the critical factor for development of the C5 nerve root palsy.
(MP4)

## Abbreviations

ACDF: Anterior Cervical Discectomy and Fusion
CRFP: Carbon fibre reinforced polymer
CSF: Cerebral spinal fluid
CSM: Cervical spondylotic myelopathy
ENT: Ear Nose and Throat
Level: e.g. level C5, only describes vertebral body C5
MMT: Manual Muscle Testing
n.s.: not significant
OPLL: Ossification of Posterior Longitudinal Ligament
OR: Odds Ratio
Segment: e.g. segment C4/5, incl. vertebral body C4, disc C4/5 and vertebral body C5
spEMG: spontaneous electromyography monitoring
TceMEPs: transcranial electrical motor-evoked potential
